# Chartered Society of Massage and Remedial Gymnastics

**Published:** 1920-10-09

**Authors:** 


### Chartered Society of Massage and Remedial Gymnastics.

The new Chartered Society has speedily set its house in order, and its register is already open to such as are qualified under various headings. This is a sign of great energy on the part of the officials responsible. The regulations drawn up to govern the admission of masseurs and masseuses to the Register show considerable skill. There is a double danger in forming a register of professional persons The first is that of discouraging applicants by making the qualification too hard of attainment. If this is done the registered members become a mere clique and the profession is no better off than before. But, on the other hand, if the admission is made too easy, it throws discredit on the whole body of the registered and may discourage highly qualified persons from applying.

The Chartered Society of Massage has very wisely avoided the pitfall of the "bona fide." In fact the "bona-fide" masseuse will not do at all. There was little difficulty in singling out from the immense crowd of masseuses who practise, or have practised, this art the ladies who have devoted serious study under reasonably good conditions to acquiring it. These and these alone are to be admitted to the register of the Chartered Society during the period of grace. All members of the I.S.T.M. and of the I.M.R.G. satisfy this condition and can be placed on the register automatically as well as certificate holders of the I.S.T.M. Certificated masseurs, who were pupils of Dr. Fletcher Little, the London School of Massage, the National Hospital, and certain other schools, prior to June 1916, are also eligible during the ensuing year, but applicants must satisfy the Council as to their professional efficiency and personal character.

For certain other applicants Test Examinations have been instituted, the first of which will be held in Manchester on October 26. These include associates of the Institute under By-law 4; certificated masseurs other than those above specified; and almo masseuses who come under By-law 4, with certificate holders from the Physical Training Colleges recognised by the Council who have passed in massage.

The interests of masseurs and masseuses are sufficiently safeguarded by these regulations, which will exclude none who have gone through a reputable course of training for their profession. At the same time the public is protected by the exclusion of men and women from the register who have merely gained a smattering of a very difficult art and on the strength of this have competed with professionals. Where there is any doubt the test examination serves a most useful purpose, and none who shrink from displaying their skill under the eye of competent observers can feel aggrieved if they are excluded from the register of the Society.

We need hardly emphasise the importance to all concerned of applying at once for registration. This is such an opportunity as the profession has long ardently desired.

